# Genome-Wide Survey and Comparative Analysis of LTR Retrotransposons and Their Captured Genes in Rice and Sorghum

**DOI:** 10.1371/journal.pone.0071118

**Published:** 2013-07-29

**Authors:** Shu-Ye Jiang, Srinivasan Ramachandran

**Affiliations:** Temasek Life Sciences Laboratory, National University of Singapore, Singapore; Ben-Gurion University, Israel

## Abstract

Long terminal repeat (LTR) retrotransposons are the major class I mobile elements in plants. They play crucial roles in gene expansion, diversification and evolution. However, their captured genes are yet to be genome-widely identified and characterized in most of plants although many genomes have been completely sequenced. In this study, we have identified 7,043 and 23,915 full-length LTR retrotransposons in the rice and sorghum genomes, respectively. High percentages of rice full-length LTR retrotransposons were distributed near centromeric region in each of the chromosomes. In contrast, sorghum full-length LTR retrotransposons were not enriched in centromere regions. This dissimilarity could be due to the discrepant retrotransposition during and after divergence from their common ancestor thus might be contributing to species divergence. A total of 672 and 1,343 genes have been captured by these elements in rice and sorghum, respectively. Gene Ontology (GO) and gene set enrichment analysis (GSEA) showed that no over-represented GO term was identified in LTR captured rice genes. For LTR captured sorghum genes, GO terms with functions in DNA/RNA metabolism and chromatin organization were over-represented. Only 36% of LTR captured rice genes were expressed and expression divergence was estimated as 11.9%. Higher percentage of LTR captured rice genes have evolved into pseudogenes under neutral selection. On the contrary, higher percentage of LTR captured sorghum genes were under purifying selection and 72.4% of them were expressed. Thus, higher percentage of LTR captured sorghum genes was functional. Small RNA analysis suggested that some of LTR captured genes in rice and sorghum might have been involved in negative regulation. On the other hand, positive selection has been observed in both rice and sorghum LTR captured genes and some of them were still expressed and functional. The data suggest that some of these LTR captured genes might have evolved into new gene functions.

## Introduction

A transposable element (TE) is a mobile genetic sequence that can transpose itself from a genomic position to another site within a genome. TEs can be grouped into two classes. Class I TEs are named as retrotransposons and they translocate themselves through an RNA intermediate mode by a “copy and paste” mechanism. Class II TEs are called as DNA transposons and they move themselves directly by a “cut and paste” mechanism through a DNA intermediate mode. Based on the transposition mechanism and structure, class I TEs can be further divided into two major subclasses including long terminal repeat (LTR) and non-LTR retrotransposons. LTR retrotransposons exist in all analyzed eukaryotic genomes [1]. They are highly prevalent and are ubiquitous components of plant genomes [2], [3]. LTR retrotransposons are characterized by two direct LTRs that flank their internal coding regions and/or other sequences. Two enzymes (gag and pol) are required for active retrotransposition. LTR retrotranspositions that encode these enzymes are called autonomous elements and those which lack these enzyme genes are termed non-autonomous elements. Due to their “copy and paste” mechanism of transposition, their copy numbers tend to be increased, while these elements are active and as a result, their host genome sizes gradually increase. In fact, the differences in genome size observed in the plant kingdom are accompanied by variations in LTR retrotransposon content [2]. The size of the *Arabidopsis* genome is around 125 Mb and contains 20 to 25 Mb of retrotransposons (16–20%) [4], whereas the maize genome is around 2,045 Mb (excluding gaps) and is occupied by more than 1,800 Mb of retrotransposons (88%) [5]. Majority of them are LTR retrotransposons. Thus, LTR retrotransposons might play important roles in genome expansion.

LTR retrotransposons contribute to not only the increase of genome size, but also deletion of genome sequences. In rice, more than 190 Mb of LTR retrotransposon sequences have been deleted from the rice genome in the last 8 million years [6]. The deletion might have occurred after the recombination events either between two LTRs of a retrotransposon, which might lead to the formation of solo-LTRs, or between direct repeats anywhere in the sequence of the element, leading to internal deletions [2]. Therefore, LTR retrotransposons significantly contribute to the growth of a genome and its evolution.

TEs might cause gene mutations after transposing into gene or promoter regions. Evidence also suggests that these elements play vital roles in gene expansion, diversification and evolution. For example, in maize, rice and Arabidopsis, mutator-like transposable elements (MULEs) were shown to carry fragments of cellular genes [7]. Lots of retrogenes were also identified in both animals and plants [8]–[10]. Insertions of small size of LTR retrotransposons into or near genes can alter both structures and expression of genes [11]. LTR retrotransposons can also transduce host sequences to generate a new chimeric gene [12]. In maize, more than 400 genes have been identified as LTR retrotransposon captured genes [5], [13]. However, in most of completely sequenced genomes, LTR captured genes are yet to be identified and characterized. In this study, we first carried out a genome wide identification of all LTR retrotransposons using the publicly available rice and sorghum genomes. We then identified their captured genes based on the latest versions of rice and sorghum gene annotation systems as well as their expansion modes. These LTR captured genes were subsequently submitted to gene set enrichment analysis and Pfam (http://pfam.sanger.ac.uk/) domain searches for further characterization. We also compared and evaluated protein divergence of these LTR captured genes by *Ka/Ks* analysis (where *Ka* = nonsynonymous substitutions per site, and *Ks* = synonymous substitutions per site). In addition, expression profiling and divergence were also carried out to further evaluate their functional divergence after expansion. Our data showed that LTR retrotransposons significantly contributed to genome and gene expansion in the rice and sorghum genomes. A total of 672 and 1343 captured genes by LTR retrotransposons have been identified in the rice and sorghum genomes, respectively. In sorghum, more than 70% of these genes were expressed whereas only around one third of LTR captured rice genes were expressed. Considerable LTR captured genes in sorghum also exhibited functional constrains and might be still functional. However, high percentage of LTR captured rice genes might have involved into pseudogenes with undetectable expression and less functional constraints as shown by *Ka/Ks* analysis.

## Results

### Genome-wide identification of LTR retrotransposons and their chromosomal distribution in rice, sorghum and maize

**Figure 1:**
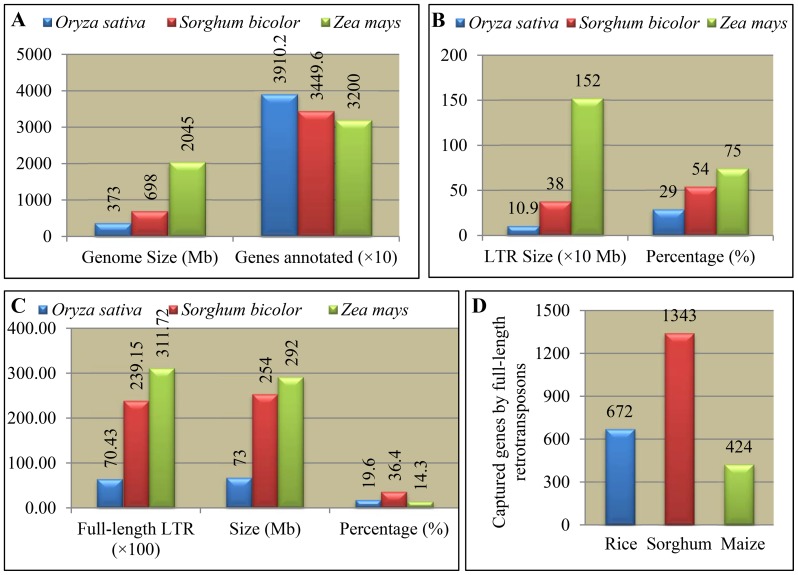
LTR retrotransposons in the rice, sorghum and maize genomes. (**A**) A general information of the rice, sorghum and maize genomes including their genome size and annotated genes. The rice genome size and annotation were based on the release 7 of pseudomolecules (http://rice.plantbiology.msu.edu/). The genome size of sorghum and maize as well as their annotation were estimated according to Paterson et al. (2009) [15] and Schnable et al. (2009) [5]. (**B**) The occupied genome size of LTR retrotransposons and its percentage in the whole genome in rice, sorghum and maize. (**C**) Genome-wide identification of full-length LTR retrotransposons and their occupied genome size as well as percentages in rice, sorghum and maize. (**D**) LTR captured genes in rice, sorghum and maize.

The rice, sorghum and maize genomes have been sequenced [5], [14], [15] with 373, 698 and 2,045 Mb, respectively, in their genome sizes and they encode around 39,102, 34,496 and 32,000 protein-coding genes (Figure 1A). To execute the LTR_Finder [3], RepeatMasker (http://www.repeatmasker.org/) and Hidden Markov Model (HMM) (HMMER 2.3.2, http://hmmer.janelia.org/) search programs, we have identified all putative solo or full-length LTR retrotransposons in both the rice and sorghum genomes. For the LTR retrotransposons in maize, data from Baucom et al. (2009) [13] were used in this study. These three genomes contained at least 109 (rice), 380 (sorghum) and 1,520 (maize) Mb of LTR retrotransposons, accounting for 29% (rice), 54% (sorghum) and 75% (maize) of their corresponding genomes (Figure 1B). Among them, we have identified a total of 7,043, 23,915 and 31,172 full-length LTR-retrtransposons in rice, sorghum and maize, respectively (Figure 1C; Table S1 and S2). These full-length LTR retrotransposons occupied 73, 254 and 292 Mb of genome sequences, accounting for 19.6%, 36.4% and 14.3% of the total sequences in these three genomes (Figure 1C). Although 75% of maize genome consists of LTR retrotransposons, only 14.3% of them are full-length elements, suggesting that high rates of recombination or deletion events might have occurred after retrotransposition. Totally, we have identified 672, 1,343 and 424 LTR captured genes in rice, sorghum and maize, respectively (Figure 1D; Table S3 and S4). Thus, although majority of the maize genome consists of LTR retrotransposons, their captured genes are limited. Therefore, here we mainly focus on both rice and sorghum full-length LTR retrotransposons and their captured genes.

**Figure 2:**
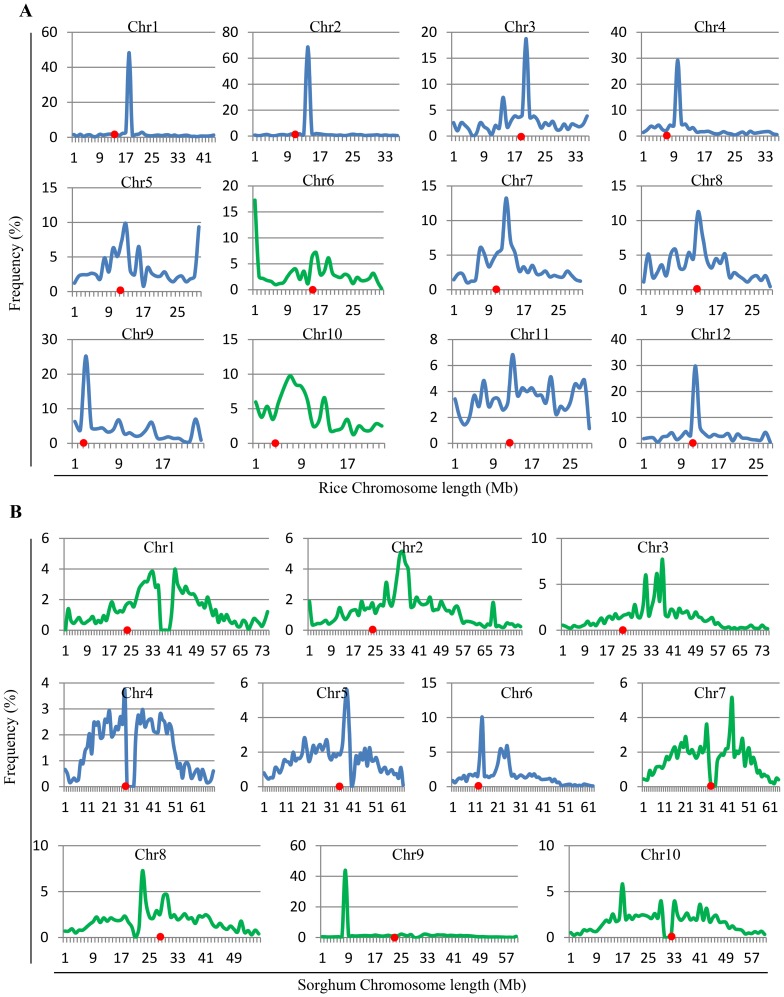
Chromosomal distributions of full-length LTR retrotransposons in the rice and sorghum genomes. Density distributions are based on the physical positions of corresponding LTR retrotransposons. X-axis indicates chromosomal positions (Mb). Y-axis indicates retrotransposon density (the percentage of total number of retrotransposons). (**A**) and (**B**) show the distributions of retrotransposons in the rice and sorghum genomes, respectively. Centromere positions are marked with red dots on each chromosome. Blue lines indicate high percentages of full-length LTR retrotransposons near chromosome centromere regions and green curves indicate that high percentages of retrotransposons are not located near centromere regions.

In maize, LTR retrotransposons are found to be most abundant in centromeric regions [13]. To figure out differences in their genome organization of full-length LTR retrotransposons between rice and sorghum, we analyzed their distributions on each chromosome based on their physical positions (Figure 2). Similar to the genome dispersal in maize, rice full-length LTR retrotransposons are also abundant near centromeric regions in 10 out of 12 chromosomes (blue curves in Figure 2A). However, in chromosomes 6 and 10, low density of retrotransposons was observed near centromeric regions (green curves in Fgure 2A). Similar results were observed in 7 out of 10 sorghum chromosomes (green curves in Figure 2B). In the sorghum genome, higher density of retrotransposons were located near centromeric regions only in chromosomes 4, 5 and 6 as indicated by blue curves in Figure 2B.

### LTR retrotransposon mediated expansion of genes in rice and sorghum

**Figure 3:**
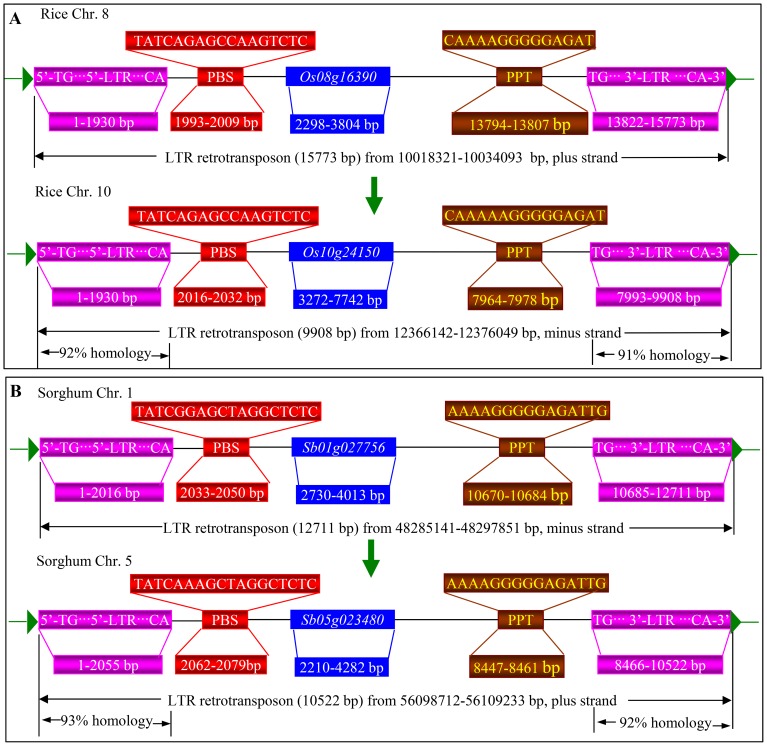
LTR retrotransposon mediated gene expansion in rice and sorghum. (**A**) and (**B**) Examples show the expansion of full-length LTR captured genes in rice and sorghum, respectively. Pink boxes indicate predicted 5′-LTR (left) and 3′-LTR (right) as well as their length. Red boxes show PBSs and their sequences as well as their positions. Brown boxes show PPTs and their sequences as well as their positions. Blue boxes indicate LTR captured genes and their positions. Green arrowheads indicate the start and end positions of a LTR retrotransposon.

Retrotransposons are among the most abundant mobile genetic elements found in plant genomes and transposed through RNA intermediate transposition. Among a total of 7,043 rice full-length LTR retrotransposons, 1,318 of them were detected to encode 1,343 non-TE proteins. This finding suggested that LTR retrotransposon mediated transductions might have contributed to the expansion of rice genes. One of such expansions has been shown in Figure 3A. The gene *LOC_Os08g16390* was annotated to encode a zinc knuckle family protein by the MSU rice genome annotation project (http://rice.plantbiology.msu.edu/index.shtml). It was located within a 15,773 bp LTR retrotransposon identified in this study. Another identified 9,908 bp LTR retrotransposon have the identical sequences of primer binding sites (PBS) and similar polypurine tract (PPT) when compared with the 15,773 bp LTR retrotransposon; they also shared 92% and 91% homology in their 5′-LTR and 3′-LTR, respectively (Figure 3A). Another gene *LOC_Os10g24150* was located in the 9,908 bp retrotransposon (Figure 3A), sharing more than 84% of identities in their protein sequences. Similarly, in sorghum, the 12,711 bp LTR captured an annotated gene *Sb01g027756*, encoding a universal minicircle sequence binding protein. The retrotransposition of this LTR retrotransposon resulted in the birth of the 10,522 bp LTR retrotransposon and thereby the expansion of the gene *Sb05g023480* (Figure 3B). These two retrotransposons showed the similar PBS sequences and the exact PPT sequences; their 5′- and 3′-LTR regions had 93% and 92% homology, respectively. Two LTR captured genes also showed 89% identities in their protein sequences.

### Comparison of functional specificities of LTR captured genes between rice and sorghum

**Figure 4:**
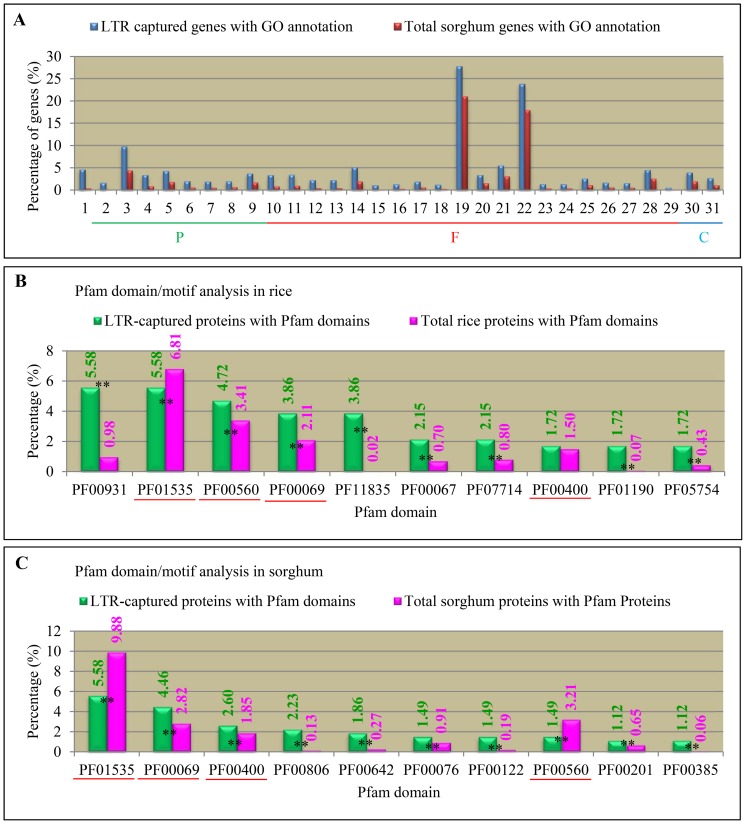
Gene set enrichment analysis and Protein domain analysis in LTR retrotransposon captured genes. (**A**) Gene set enrichment analysis in sorghum. Blue and red columns indicate the percentages of this GO term in all LTR captured sorghum proteins and in total annotated proteins, respectively. The percentage is calculated as the frequency of the total numbers of each GO term in all LTR captured proteins with GO term assigned or in all annotated proteins with GO term assigned. “P”, “F” and “C” in (**A**) indicate GO three categories biological process, molecular function and cellular component, respectively. GO term annotation in (**A**) was shown below: 1, DNA integration; 2, reproductive cellular process; 3, DNA metabolic process; 4, RNA-dependent DNA replication; 5, DNA replication; 6, multicellular organism reproduction; 7, reproductive process in a multicellular organism; 8, chromatin assembly or disassembly; 9, chromatin organization; 10, RNA-directed DNA polymerase activity; 11, DNA polymerase activity; 12, aspartic-type endopeptidase activity; 13, aspartic-type peptidase activity; 14, nucleotidyltransferase activity; 15, ribonuclease H activity; 16, endonuclease activity, active with either ribo- or deoxyribonucleic acids and producing 5′-phosphomonoesters; 17, chromatin binding; 18, endoribonuclease activity, producing 5′-phosphomonoesters; 19, nucleic acid binding; 20, endopeptidase activity; 21, peptidase activity; 22, hydrolase activity; 23, di-, tri-valent inorganic cation transmembrane transporter activity; 24, endoribonuclease activity; 25, nuclease activity; 26, endonuclease activity; 27, ribonuclease activity; 28, peptidase activity, acting on L-amino acid peptides; 29, zinc ion transmembrane transporter activity; 30, chromosomal part; 31, chromatin. (**B**) and (**C**) Pfam domain/motif analysis in rice and sorghum, respectively. Green and pink columns indicate the percentages of this domain in the LTR captured protein and in total proteins, respectively. Commonly detected over- or under- represented domains in rice and sorghum are highlighted with pink lines. Two stars indicate statistically significant differences at P value <0.01. PF00067, oxidoreductase activity, acting on paired donors, with incorporation or reduction of molecular oxygen; PF00069, protein phosphorylation; PF00076, nucleic acid binding; PF00122, nucleotide binding; PF00201, transferase activity, transferring hexosyl groups; PF00385, ‘chromo’ (CHRromatin Organisation MOdifier) domain; PF00400, WD domain, G-beta repeat; PF00560, protein binding; PF00642, nucleic acid binding; PF00806, RNA binding; PF00931, apoptosis; PF01190, Pollen proteins Ole e I like; PF01535, PPR repeat; PF05754, Domain of unknown function (DUF834); PF07714, protein phosphorylation; PF11835, Domain of unknown function (DUF3355.

To survey the difference in functional specificities of LTR captured genes between rice and sorghum, we performed Gene Ontology (GO) analysis on these genes. We have identified GO terms in three categories including biological process (P), molecular function (F), and cellular component (C) [16]. Subsequently, we identified over-represented GO terms by gene set enrichment analysis (GSEA, see Methods). To our surprise, no over-represented GO term was identified when all LTR captured rice genes were subjected to GSEA. For LTR captured sorghum genes, we have identified 31 GO terms, which showed over-representation when compared with all annotated sorghum genes (Figure 4A). For GO terms in biological process, those genes with functions in DNA/RNA metabolism and chromatin organization were over-represented. For GO terms in molecular functions, enzymes or proteins related to DNA/RNA metabolism and/or chromatin binding were over-represented. For GO terms in cellular component, their encoded proteins were mainlyassociated with chromosomal part and chromatin.

To further investigate functional specificities of these LTR captured genes between rice and sorghum, we carried out the Pfam domain/motif searches by submitting those proteins deduced from all LTR captured genes into the Pfam database (http://pfam.sanger.ac.uk/). After the searches, 10 domains/motifs in either rice or sorghum were selected which were most frequently detected in their captured proteins for further analysis (Figure 4 B and C). Such an analysis revealed four domains/motifs that were commonly present in both rice and sorghum. These domains/motifs are with IDs PF01535 (Pentatricopeptide repeat), PF00560 (Leucine Rich Repeat), PF00069 (Protein kinase domain), and PF00400 (WD domain, G-beta repeat). The domain with ID PF01535 was under-represented in both rice and sorghum. Other domains with IDs PF00069 and PF00400 showed over-representation in both rice and sorghum. For the remaining domain with ID PF00560, no significant difference was observed when compared with the proportion of this domain to the total number of rice proteins; however, this domain was over-represented in sorghum. Besides the commonly detected 4 domains, we have also detected 6 other domains in either rice or sorghum and all these domains were over-represented in corresponding plants.

### Protein divergence and pseudogenes after expansion by LTR retrotransposons

**Figure 5:**
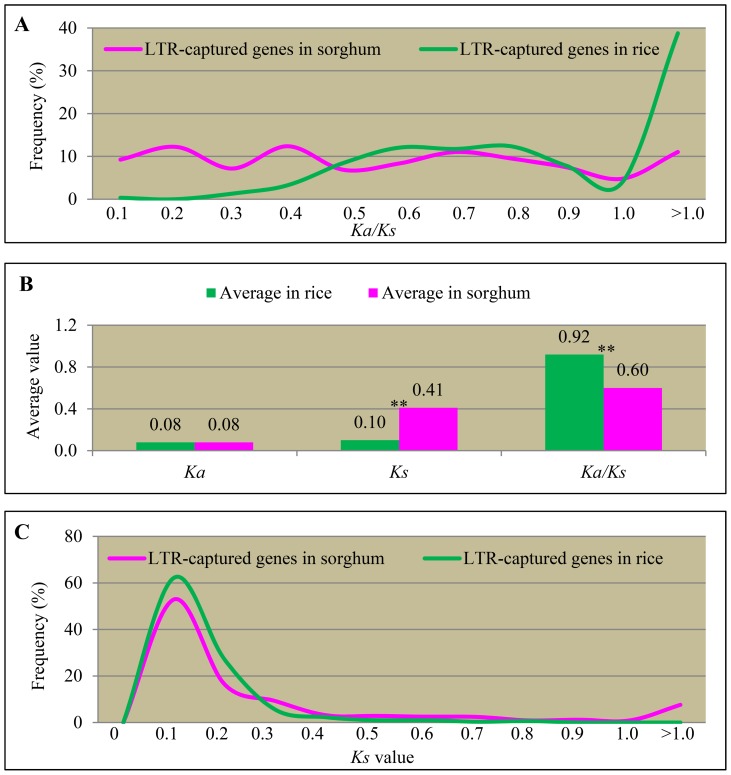
Distribution of *Ka*, *Ks* and *Ka/Ks* values of LTR captured gene pairs in rice and sorghum. (**A**) The distribution curves of *Ka/Ks* values for LTR captured gene pairs in rice (green curve) and sorghum (pink curve). (**B**) Average values of *Ka*, *Ks* and *Ka/Ks* in rice (green bars) and sorghum (pink bars). (**C**) The distribution curves of *Ks* values for LTR captured gene pairs in rice (green curve) and sorghum (pink curve).

Since considerable numbers of genes have been expanded by LTR retrotransposons, we were interested to find out whether these expanded descendants are still functional or have evolved into pseudogenes. To test their protein divergence, we analysed nonsynonymous substitutions per site (*Ka*) and synonymous substitutions per site (*Ks*) and their ratios for each expanded pairs in both rice and sorghum. In rice, high percentage of expanded pairs showed *Ka/Ks* >1 (green curve in Figure 5A) with an average of *Ka/Ks* ratio at 0.92 (Figure 5B) and very low percentage of pairs were at *Ka/Ks* ≤0.5. In sorghum, the frequency of *Ka/Ks* distribution was more even (Figure 5A) with an average of *Ka/Ks* ratio at 0.60 (Figure 5B). These data suggested that higher percentage of expanded genes in rice might have evolved into pseudogenes when compared with sorghum expanded genes by LTR retrotransposons. To further analyse the difference in their evolutionary rates between rice and sorghum, we compared their *Ka* and *Ks* values separately. We found that their average *Ka* values between rice and sorghum were similar (Figure 5B). However, they exhibited significant differences in their average *Ks* values with only 0.01 in rice and up to 0.41 in sorghum (Figure 5B). The frequency of *Ks* value distributions in rice and sorghum also showed that significantly higher percentage was centred at *Ks* = 0.1 (Figure 5C). As a result, they showed significant differences in their *Ka/Ks* distributions.

**Figure 6:**
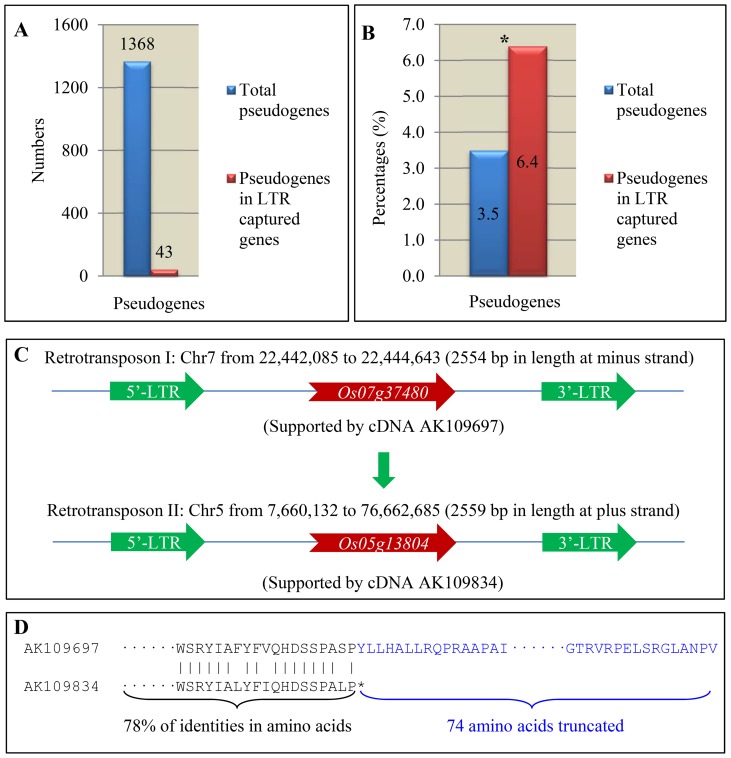
Pseudogenes in rice LTR retrotransposon captured genes. (**A**) Total of pseudogenes identified from the version 7 of rice genome pseudomolecules (blue column) and LTR captured genes (red column), respectively. (**B**) Pseudogene percentages in total of annotated genes in the version 7 of rice genome (blue column) and in LTR captured genes (red column), respectively. (**C**) An example of expansion and evolution of a pseudogene in rice. (**D**) An expanded gene is truncated in its encoded protein, which was supported by corrsponding full-length cDNA sequence and is regarded as a pseudigene.

As our preliminary analysis suggested higher ratio of pseudogenes in rice LTR captured genes, we further analysed the contribution of LTR retrotransposons to the evolution of rice pseudogenes. Using the release 7 version of rice genome annotation dataset, we have identified a total of 1,368 annotated genes with typical features of pseudogenes such as the presence of frameshifts or premature translational stop codons, accounting for 3.5% of total annotated non-TE genes (Figure 6 A and B). Among them, up to 6.4% (43 putative pseudogenes) were LTR captured genes (Figure 6 A and B). Statistical data suggest a significantly higher contribution of LTR retrotransposons to the evolution of rice pseudogenes. One of the examples was the pseudogene with annotated locus *LOC_Os05g13804*. This gene was supported by a full-length cDNA with accession number AK109834 (http://cdna01.dna.affrc.go.jp/cDNA/) and was expanded by a retrotransposition event from its parental gene *LOC_Os07g37480* (supported by full-length cDNA AK109697). In the event, the retrotransposon I located on chromosome 7 was retrotransposed into chromosome 5 and gave birth to the retrotransposon II and its captured gene was also expanded (Figure 6C). After expansion, the gene *LOC_Os05g13804* might have a target for mutations which might have led to premature translational stop codons, and as result, encoded a truncated protein as shown in Figure 6D.

### Expression profiling and divergence of LTR captured genes in rice and sorghum

**Figure 7:**
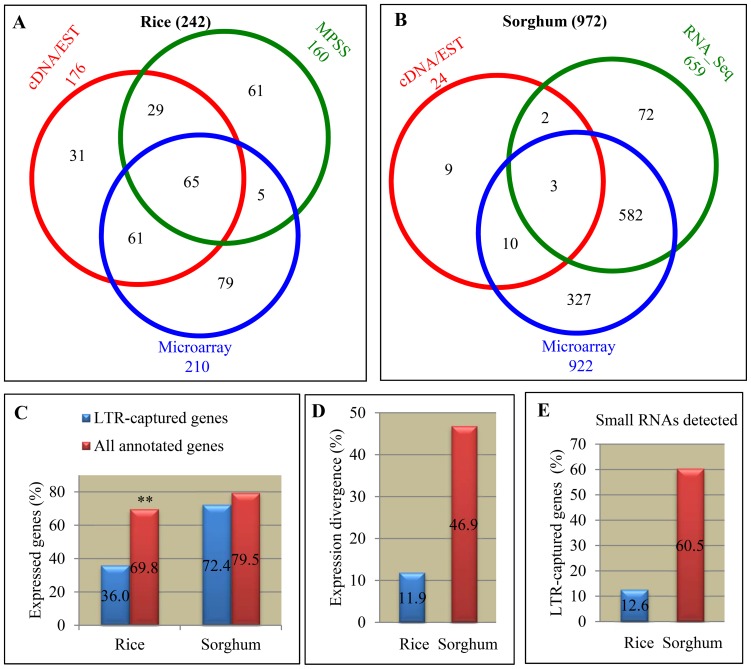
Expression and smRNA analysis. (**A**) and (**B**) A summary of expression profiling of LTR captured genes in rice and sorghum, respectively. The analysis was based on collected full-length cDNA/EST, MPSS, microarray expression data and/or RNA_Seq data. (**C**) Expression percentages of LTR captured genes and total annotated genes in rice and sorghum. (**D**) Expression divergence of genes expanded by LTR retrotransposons in rice and sorghum. (**C**) Unique and expressed small RNAs located on LTR captured genes in rice and sorghum.

As our *Ka/Ks* analysis showed that some of the expanded genes by LTR retrotransposons have evolved into pseudogenes, we further analyzed their expression profiling. We used three different expression databases to analyze their expression including cDNA/EST (Expressed Sequence Tag), microarray or RNA_seq/MPSS (Massively Parallel Signature Sequencing, see Methods for more details). In rice, we have detected 176 genes with cDNA/EST evidence, 160 and 210 genes with expression signaling in rice MPSS and microarray analysis, respectively (Figure 7A). In sorghum, only 24 genes were detected with cDNA/EST evidence due to a limited collection of this database (Figure 7B). However, we have detected 659 and 922 genes with detectable signaling in RNA_Seq and microarray datasets, respectively (Figure 7B). Since some of these genes were detected with expression in more than one database, as a result, only 36% of LTR captured rice genes (242 genes) were expressed while up to 69.8% of total annotated rice genes were expressed (Figure 7C). However, in sorghum, 72.4% of LTR captured genes showed expression, similar to the ratio where 79.5% of total annotated sorghum genes were expressed (Figure 7C).

We were also interested to find out whether an expanded gene showed expression divergence when compared with its paralogous gene. Our data showed that only 11.9% of LTR captured rice genes exhibited expression divergence in their transcript abundance (Figure 7D). However, in sorghum, up to 46.9% of LTR captured sorghum genes showed expression divergence (Figure 7D), significantly higher than that in rice. On the other hand, it is also of interest for us to know whether these LTR captured genes could be involved in small RNA (smRNA)-based gene-silencing pathway. Only unique smRNAs were selected for such analyses (see Methods), which led to the identification of a total of 85 and 812 LTR captured genes with unique and detectable smRNA loci in rice and sorghum, respectively. These genes accounts for12.6%, 60.5% of total LTR captured genes in rice and sorghum, respectively (Figure 7E).

## Discussion

### LTR retrotransposons contribute to plant genome expansion and evolution

The genome-wide identification of LTR retrotransposons in this study and others [4], [13], [15], [17] have demonstrated that the genome size of a species was directly related to the proliferation of LTR retrotransposons and significantly contributed to the genome expansion. However, it is now clear that transposition can be deleterious to the host [13]. Retrotransposition may lead to malfunction of tagged genes and can cause major genomic modification and reorganization. Thus, host genomes impose selection on LTR retrotransposons to reduce such deleterious proliferations. As a result, genome expansion is restricted. In rice, sorghum and maize, around 29%, 54% and 75% of the genome was occupied by LTR retrotransposons, respectively (Figure 1B). Accordingly, their genome size was estimated at around 373, 698 and 2,045 Mb, respectively (Figure 1A). Reasonably, maize should have evolved a mechanism to more strongly reduce the retrotransposon activity. In fact, in maize, most of LTR retrotransposons were found to be solo retrotransposons and only 19% of retrotransposons were the full-length LTR retrotransposons, which were potentially with active transposition. On the contrary, up to 62% and 66% of rice and sorghum retrotransposons are full-length LTR retrotransposons, respectively. Furthermore, the analysis of rice genes required for the replication of LTR retrotransposons indicated that most of these genes were under strong purifying selection and were still functional [13]. We also investigated the expression profiling of 11,399 rice genes encoding retrotransposon proteins and at least 1,700 of them showed active expression. All these data suggested that some of these full-length LTR retrotransposons are still active and might contribute to increase of host genome size.

### Dissimilarities of LTR retrotransposons between rice and sorghum

Previous studies showed that rice diverged from maize and sorghum 50–70 million years ago (Mya) [18]. Sorghum and maize plants were estimated to have diverged from each other about 11.9 Mya [19]. After the divergence from each other, the activities of their LTR retrotransposons varied gradually and these elenebts transposed with different rates. As a result, these three species have evolved with total different sizes of genomes during long evolutionary history. On the other hand, sorghum is also different from rice with respect to distribution of full-length LTR retrotransposons in their chromosomes (Figure 2). Most of sorghum full-length LTR retrotransposons are not enriched in centromere regions. The comparison of centromeric retrotransposons from rice, maize, and barley revealed several highly conserved motifs [20] and maintained its centromeric specificity for >50 MY [21]. Thus, the dissimilarity in chromosome distribution could be due to the discrepant retrotransposition and insertion during and after divergence from their common ancestor. During long evolution history, the differential retrotransposition and insertion resulted in different percentages of full-length LTR retrotransposns and their captured genes. Thus, the dissimilarity might have also contributed to the species divergence and evolution.

### Functions of LTR captured genes in rice and sorghum

Many genes captured by TEs have been reported. Genes captured by non-LTR retrotransposons were designated as retrogenes [22]–[24]. Mutator-like transposable element (MULE) captured genes were designated as Pack-MULEs [7]. In rice, both retrogenes and Pack-MULEs account for the majority of TE-captured genes [10], [25], [26]. In maize, majority of TE-captured genes were from *Helitron* element [5]. In sorghum, majority of class I and II TEs are LTR retrotransposons and CACTA element, respectively [5]. Based on our analysis, CACTA-captured genes should be less than 600 members. In this study, we have identified 672 and 1,343 LTR captured genes in rice and sorghum, respectively. Thus, majority of TE captured genes in sorghum might be from LTR retrotransposons. Therefore, different species showed difference in TE-mediated gene expansion.

How did these genes evolve after expansion? In rice, a considerable number of retrogenes and Pack-MULEs might have been under selective constraint and many of them were still functional [10], [25]. Chimeric genes were frequently observed in rice retrogenes and Pack-MULEs [10], [25], providing resources for new gene functions. However, for LTR captured genes in rice, majority of them are under non-purifying selection, which might be related to the preferred integration into centromere regions. The centromere regions of most eukaryotic organisms could have evolved rapidly [27]. Higher percentages of rice full-length LTR retrotransposons were located on centromeric regions and their captured genes might also evolve more rapidly, which were implied by the *Ka/Ks* analysis (Figure 5). Only 36% of LTR captured rice genes were expressed (Figure 7C) and expression divergence was estimated as 11.9%. Thus, higher percentage of rice LTR captured genes might have evolved into pseudogenes (Figure 6). On the contrary, higher percentage of sorghum LTR retrotransposon captured genes was under purifying selection and up to 72.4% of LTR captured genes were expressed (Figure 7C). Thus, higher percentage of LTR captured genes was still functional in sorghum. On the other hand, positive selection has been observed in both rice and sorghum and some of them were still expressed and functional. The data suggest that these LTR captured genes might have evolved into new gene functions.

## Materials and Methods

### Genome sequence and annotation databases

The release 7 of the rice pseudomolecules and genome annotation data were downloaded from the MSU (Michigan State University) rice genome annotation project [28] (http://rice.plantbiology.msu.edu). Total of 373,245,519 bp of non-overlapping rice genome sequences were assembled from the 12 rice chromosomes and 39,102 non-TE genes were annotated. For sorghum, the genome sequences of v1.0 release of Sbi1 assembly were downloaded from the Phytozome sorghum database [15] (http://www.phytozome.net/sorghum). In this database, total of 697,578,683 bp of genome sequences from the 10 sorghum chromosomes were assembled and 34,496 genes/loci were annotated.

### Identification of LTR retrotransposons and their captured genes in rice and sorghum

The LTR_FINDER program [3] was used to identify the full-length LTR retrotransposons in the rice and sorghum genomes by default setting. The program detected the elements based on the presence of LTRs, target site repeats (TSRs), PBSs, PPT, TG … CA box, and coding genes for reverse transcriptase (RT), integrase (IN) and RNaseH (RH). The maximum distance between LTRs was set to 20 Kb and the maximum LTR length was set to 3.5 Kb. Both “Output score threhold” and “extension cutoff” were set to 6.0 and 0.8, respectively. Under these settings, the maximum length of identified LTR retrotransposons is 26,529 bp and 26,390 bp in the rice and sorghum genomes, respectively. RepeatMasker 3.3.0 was downloaded from the website http://www.repeatmasker.org/ and was used for screening interspersed repeats and low complexity DNA sequences. Solo LTRs are created by recombination between two full-length LTRs. To identify solo LTRs, LTRs identified by LTR_FINDER and RepeatMasker were first clustered based on their sequence similarity. The represented LTRs were used to generate a profile HMM using HMMBuild from the HMMER package (HMMER 2.3.2, http://hmmer.janelia.org/). HMMSearch was used to search for HMMs against the entire genome to identify potential LTRs, including solos. The threshold of E-value cutoff was set up as 1.0e-9, which was determined based on the best recovery of known solo LTRs. Each identified solo-LTR showed at least 70% of identity to the query sequence. Genes located within a full-length LTR retrotransposon were regarded as LTR captured genes.

### Pfam domain searches and analysis

Protein sequences deduced from LTR captured genes were downloaded from the above mentioned annotation databases. These sequences were then submitted to the Pfam database (http://pfam.sanger.ac.uk/) for domain detection with the default setting. Top 10 frequently observed domains were collected for each organism. The percentage of a collected domain presented in the LTR captured proteins was compared with that in the whole annotated proteins. Pearson's *χ^2^* test was used to determine if a domain was over- or under- represented.

### GO annotation and gene set enrichment analysis (GSEA)

GO annotations were downloaded for rice and sorghum from the MSU rice annotation project and the Phytozome sorghum database, respectively. More than one GO slim terms could be assigned for each gene in three functional categories including biological process (P), molecular function (F), and cellular component (C). We investigated each GO slim term category independently. To determine if a GO category was over-represented in all LTR captured rice or sorghum genes, GSEA was employed with nominal p-value <0.05 and false discovery rate (FDR) <0.25 [29].

### *Ka/Ks* estimation and identification of pseudogenes

Coding sequence and protein data for rice and sorghum were downloaded from the above mentioned annotation databases. The longest alternatively spliced form of peptides were selected for calculations. Pairs of homologous proteins were aligned with ClustalX [30], using default setting. The perl program PAL2NAL [31] was used to convert the aligned protein sequences into the corresponding coding sequences. The resulted pairwise alignments were submitted to the PAML package and the YN00 program [32] for calculating *Ka* and *Ks* values.

Pseudogenes were identified according to the description [33]. Briefly, most of pseudogenes were derived from their parental genes by duplication or transposition/retrotransposition. Pseudogenes were identified when they covered at least 70% of the parent proteins for alignment and were featured with disablements (frameshifts or premature translational stop codons).

### Expression analysis

Rice cDNA sequences were downloaded from the website http://cdna01.dna.affrc.go.jp/cDNA/ [34]. Both rice and sorghum ESTs (expressed sequence tags) as well as sorghum cDNA sequences were downloaded from the NCBI database (http://www.ncbi.nlm.nih.gov/). A gene was regarded as expressed gene if corresponding cDNAs or ESTs could be detected with 95–100% identity over 90% of the length of cDNAs or ESTs. Rice MPSS data were downloaded from the website http://mpss.udel.edu/rice/mpss_index.php [35]. An expressed gene was identified when the normalized abundance values from both the 17-bp and 20-bp signature databases were not less than 5 in the MPSS database. Rice and sorghum microarray data were downloaded from the Gene Expression Omnibus (GEO) datasets [36] (http://www.ncbi.nlm.nih.gov/geo/) with accession numbers GSE6893 and GSE36689, respectively. We determined if a gene is expressed or not according to the criteria as described by Jiang et al. (2009) [26]. Sorghum RNA_Seq data were also downloaded from the GEO datasets with accession number GSE30249 and corresponding expression analysis was carried out according to the description [37].

### Small RNAs

Rice and sorghum small RNA data were downloaded from the GEO datasets with accession number GSM803128 and GSM943193, respectively. LTR captured rice or sorghum gene sequences were submitted to BLAST searches against these two smRNA datasets with 100% identities over the full-length smRNA sequences. The detected smRNAs were submitted to the secondary BLAST searches against the whole rice or sorghum genome sequences to filter these smRNAs with multiple homologous sites in the rice or sorghum genomes. Only the smRNAs with unique site in each LTR captured gene was used for further analysis.

## Supporting Information

Table S1 **Genome-wide identification of full-length LTR-retrotransposons in the rice genome.**
(XLS)

Table S2 **Genome-wide identification of sorghum full-length LRT-retrotransposons.**
(XLS)

Table S3 **LTR-retrotransposon captured genes in rice.**
(XLS)

Table S4 **LTR-retrotransposon captured genes in Sorghum.**
(XLS)
